# Immunity, Ion Channels and Epilepsy

**DOI:** 10.3390/ijms23126446

**Published:** 2022-06-09

**Authors:** Tsang-Shan Chen, Ming-Chi Lai, Huai-Ying Ingrid Huang, Sheng-Nan Wu, Chin-Wei Huang

**Affiliations:** 1 Department of Neurology, Tainan Sin-Lau Hospital, Tainan 701002, Taiwan; tschern@gmail.com; 2 Department of Pediatrics, Chi-Mei Medical Center, Tainan 71004, Taiwan; vickylai621@gmail.com; 3 Neuroscience Program, McGill University, Montréal, QC H3A0G4, Canada; huaiying3655@gmail.com; 4 Department of Physiology, College of Medicine, National Cheng Kung University, Tainan 70101, Taiwan; snwu@mail.ncku.edu.tw; 5 Institute of Basic Medical Sciences, National Cheng Kung University Medical College, Tainan 70101, Taiwan; 6 Department of Neurology, National Cheng Kung University Hospital, College of Medicine, National Cheng Kung University, Tainan 70101, Taiwan

**Keywords:** immunity, ion channel, epilepsy, seizure, inflammation

## Abstract

Epilepsy is a common chronic neurological disorder in modern society. One of the major unmet challenges is that current antiseizure medications are basically not disease-modifying. Among the multifaceted etiologies of epilepsy, the role of the immune system has attracted considerable attention in recent years. It is known that both innate and adaptive immunity can be activated in response to insults to the central nervous system, leading to seizures. Moreover, the interaction between ion channels, which have a well-established role in epileptogenesis and epilepsy, and the immune system is complex and is being actively investigated. Some examples, including the interaction between ion channels and mTOR pathways, will be discussed in this paper. Furthermore, there has been substantial progress in our understanding of the pathophysiology of epilepsy associated with autoimmune encephalitis, and numerous neural-specific autoantibodies have been found and documented. Early recognition of immune-mediated epilepsy is important, especially in cases of pharmacoresistant epilepsy and in the presence of signs of autoimmune encephalitis, as early intervention with immunotherapy shows promise.

## 1. Introduction

Epilepsy is a chronic brain disorder that causes chronic, recurrent seizures as part of its clinical presentation. It is estimated that between 1% and 1.5% of the global population experiences at least one seizure in their lifetime. Although the techniques and technologies used in brain imagery and in neurophysiological research have undergone substantial development in recent years, some of the etiologies of epilepsy have not yet been identified, and the mechanisms of epilepsy are still not fully understood. Consequently, the treatment of epilepsy is not always satisfactory. It is estimated that 30% of patients with epilepsy suffer from pharmacoresistant epilepsy [1,2]. One of the unmet challenges is the difficulty of explaining epileptogenesis. The problem stems from the fact that the antiepileptic drugs (AEDs) currently used to treat epilepsy are basically not disease-modifying drugs; instead, they are antiseizure drugs that are designed to reduce the frequency of seizures but not to alter epileptogenesis [3].

Epilepsy is a multifaceted condition with complex etiologies, including genetic, toxic, and metabolic causes; infection; and structural lesions in the brain. Another possible cause has come to light recently, as the investigation of the role of immune mechanisms in the pathogenesis of seizures has gained momentum over the past two decades. Furthermore, the classification of seizures and epilepsies published in 2017 by the International League Against Epilepsy (ILAE) included a novel immune-mediated origin as one of the six etiologies of epilepsy [4]. It is known that both innate and adaptive immunity can be activated in response to central nervous system (CNS) insults, which, in turn, could lead to seizures [5]. Several neural-specific autoantibodies have been identified, such as the anti-Hu antibody in patients with paraneoplastic encephalomyelitis, the anti-Ma1 antibodies associated with paraneoplastic neurological syndromes, the anti-Ma2 antibodies associated with limbic encephalitis, and the anti-N-methyl-D-aspartate (NMDA) receptor antibodies in patients with limbic encephalitis [6,7,8,9,10,11,12]. Additionally, a retrospective population-based study in the US revealed a fourfold increase in the risk of epilepsy among patients with autoimmune disease [13]. These findings shed light on the role of immunity in the pathogenesis of epilepsy. In addition, some studies have suggested that the mammalian target of the rapamycin (mTOR) pathway plays a key role in the proper development of neural networks and that it is involved in epileptogenesis triggered by both genetic and acquired factors [14,15,16].

The role of ion channels in epilepsy and epileptogenesis is an active focus of current research, and the alteration of the ion channels involved in epileptogenesis has been established in numerous studies [17,18,19,20,21]. It has been further suggested that some ion channels are associated with altered immunological/inflammatory responses involved in the generation of epilepsy [22,23] and in immune-mediated epilepsy.

To address the difficulty of treating epilepsies of unknown etiology and epilepsies that are refractory to standard antiseizure medications, the identification of immune-mediated epilepsy may prove beneficial, and the early administration of immunotherapy may produce favorable clinical outcomes [24]. In this review, we will discuss recent research on immunity activation and neuroinflammation, as well as the neuronal autoantibody targeting of specific cells, the implications for epileptogenesis, the impact on the progression of the disease per se, the role of ion channels, and the interaction with immune response.

## 2. Scope of Review

A literature search was conducted using the following academic databases: PubMed and MEDLINE. The search criteria included peer-reviewed journal articles, including original articles, case reports, clinical trials, reviews, meta-analyses, reviews, and systematic reviews. The main search terms used were “immunity” OR “immunological response” AND “ion channel” AND “epilepsy” OR “seizure”. Additional key search terms included “inflammation”, “neuronal excitability”, “autoimmune encephalitis”, and “autoimmune epilepsy”. Searches were restricted to articles in the English language. Articles published between 1 Jan 1995 and 31 Mar 2022 were evaluated. Initial screening of the search results involved inspection of the articles’ titles and abstracts. The full text of articles considered for inclusion was then screened. Articles were excluded if, upon inspection, they were found to not contain information regarding the interaction between immunity and seizure/epilepsy or immunity and ion channels. Letters/case study articles and others were excluded during full-text screening if they did not meet the above criteria. Articles that did not address the interaction between immunity and seizure/epilepsy or immunity and ion channels were excluded. After removing duplicates and adequate screening, the search resulted in 174 articles. The progression in number of publications mentioned in this review was remarkable (articles from 1980–1990: 1; 1990–2000: 12; 2000–2010: 44; 2010–2022: 117).

## 3. Activation of Innate Immunity and Neuroinflammation

Evidence is mounting in support of the involvement of glial cells in the innate immune responses of the brain. Glial cells have long been thought to be responsible for repairing damaged brain tissue (reactive gliosis) resulting from brain insults such as infection, ischemia, and trauma. The activation of glial cells, their synthesis, and their release of inflammatory substances initiate the innate immune response [25,26,27]. In animal experiments, intracortical injection of a high concentration of lipopolysaccharide (LPS) binding to Toll-like receptor 4 (TLR4) caused the release of interleukin-1 (IL-1) from glial cells, leading to an increase in both the amplitude of the evoked field potential and in focal epileptiform activity [28]. Furthermore, microglial activation and leukocyte infiltration have been observed in the sclerotic tissue of patients with mesial temporal lobe epilepsy (TLE) [29]. Glial activation also occurs during epileptogenesis, which causes recurrent seizures and progression of the disease [29]. Finally, it has also been shown that chronic inflammation-related glial scarring, generally regarded as epileptogenic, is involved in axonal sprouting and abnormal neuronal excitability [30].

**Figure 1:**
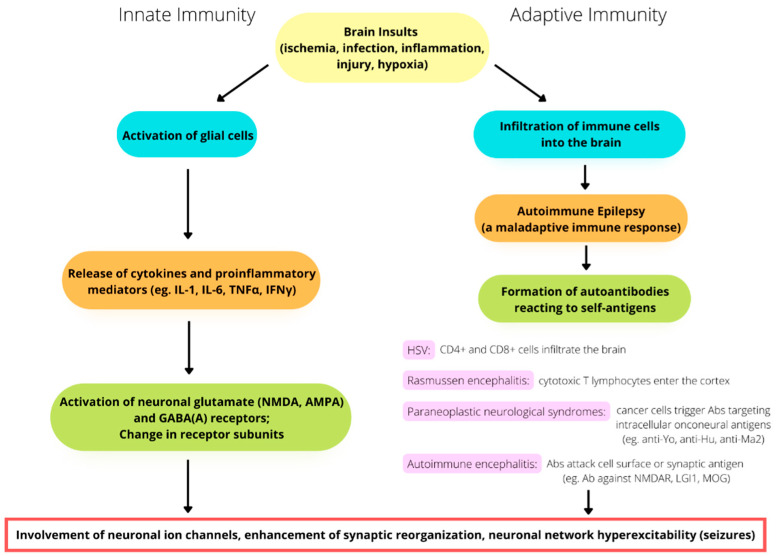
The involvement of immunity in the pathomechanism of seizure generation. Both innate and adaptive immunity alterations play a potential role in seizures and epileptogenesis.

Inflammatory cells, such as macrophages and neutrophils, have been reported to be abundant in the brain during epileptogenesis [29]. Starting with the activation of glial cells and proceeding to their release of proinflammatory mediators, the levels of cytokines and chemokines in the brain increase, activating the expression of neuronal glutamate (NMDA and α-amino-3-hydroxy-5-methyl-4-isoxazolepropionic acid, known as AMPA) receptors and neuronal type-A ϒ-aminobutyric acid (GABA_A_) receptors and altering their receptor subunits [31]. Neuroinflammatory cytokines also contribute to synaptic reorganization, causing neural network hyperexcitability (Figure 1). The cytokines IL-1α and IL-1β, in particular, exert strong proinflammatory effects. IL-lβ has been shown to reduce GABA-mediated inhibition [32] and to facilitate NMDA-receptor-mediated calcium influx [33]. Studies have also shown that intrahippocampal injection of IL-1β can prolong the duration of kainic-acid-induced seizures in adult animals [34]. Finally, following the introduction of the cytokines tumor necrosis factor alpha (TNFα), IL1β, and interferon gamma (IFNγ) into the cortical neurons of Murinae, evidence of network perturbations was detected through analysis of calcium transients [35] and animal seizure models [21].

Astrocytes, one of the four types of glial cells that have traditionally been thought to be neuron-supporting cells, also play an important role in the mechanisms of TLE. There is some evidence to suggest that the TLE mechanism acts by inducing synaptogenesis [36,37,38]. The most common pathological change found in surgical specimens from patients with pharmacoresistant TLE is hippocampal sclerosis (HS), which is characterized by the loss of pyramidal neurons and reactive astrogliosis. Astrogliosis, as a defense mechanism of the CNS, can repair tissue that has been damaged as a result of brain injury. It also produces reactive astrocytes, which can be harmful to the brain because they worsen neuroinflammation, release excitatory glutamate, and break down the blood–brain barrier (BBB), all of which increase the likelihood of seizures being generated [39,40,41]. The BBB is crucial for maintaining homeostatic control of brain functions, and a breach of the BBB changes its permeability and allows cytokines to enter the brain via the bloodstream [42,43,44]. In addition, increased binding of the 18 kDa translocator protein, which is overexpressed in activated microglia and reactive astrocytes, has been observed via positron emission tomography (PET) imaging in patients with TLE [45]. On the other hand, in children with chronic intractable epilepsy encompassing cortical dysplasia, encephalomalacia, and TLE, significant microglial activation and diffuse astrocyte proliferation have been found in cortical tissue [46]. Proinflammatory cytokines, such as IL1β and high-mobility group box protein 1 (HMGB1), are seen not only in TLE but also in other epileptic etiologies, such as cortical dysplasia [47]. This evidence highlights the role of neuronal immunity and inflammation in the pathogenesis of epilepsy.

## 4. Adaptive Immunity in Seizures and Epilepsy

Unlike innate immune responses, which are triggered by glial activation and inflammation, which, in turn, play a role in epileptogenesis, adaptive immune responses are caused by immune cells infiltrating the brain as a result of infectious or noninfectious encephalitis, trauma, or hypoxia. Autoimmune epilepsy originates with a maladaptive immune response, which results in the formation of autoantibodies reacting to self-antigens. Adaptive immunity is not only related to recurrent seizures but is also involved in the progressive degeneration of the brain. For example, in patients with Rasmussen’s encephalitis, staining for CD8 has demonstrated the large-scale infiltration of cytotoxic T lymphocytes into the cortex [48] (Figure 1).

In the case brain infection by the herpes simplex virus (HSV), proinflammatory cytokines are produced by microglia and locally infiltrating macrophages. HSV sometimes triggers the late onset of antibodies attacking NMDA receptors, leading to the development of anti-NMDA receptor encephalitis [49,50,51,52]. Chemokines are also released during HSV infection and play a role in immunity by modulating leukocyte trafficking to the focus of infection [53,54,55]. As the virus continues replicating, both CD4^+^ and CD8^+^ T lymphocytes infiltrate the brain [56,57,58]. Incidentally, HSV can escape from immune response targeting in the brain through the mediating effect of HSV-1 UL13 kinase. This kinase facilitates the evasion of HSV-1-specific CD8^+^ T cells at infection sites by downregulating the expression of CXCL9, a chemokine that attracts the CD8^+^ T cells, thereby increasing the severity and fatality of the HSV infection [59].

Another example of adaptive immunity is paraneoplastic neurological syndromes (PNSs), in which the production of antibodies is triggered by cancer cells and attack neurons. This spectrum of diseases is usually caused by antibodies targeting intracellular onconeural antigens. The pathogenesis is most likely mediated by T cells, as conspicuous cytotoxic T-cell infiltration has been found surrounding neurons in patients with anti-Yo, anti-Hu, and anti-Ma2 antibodies [60,61,62]. In the past two decades, autoimmune encephalitis (AE) has become known and has changed our approach to evaluating the etiologies of epilepsy [63,64,65] (Figure 1). AE might occur in conjunction with PNSs or not be related to cancers. The antigens in AE tend to be located on the cell surface or synapse and are targeted by antibodies, such as the antibodies against NMDA receptors, leucine-rich glioma-inactivated protein 1 (LGI1), and myelin oligodendrocyte glycoprotein (MOG). On the other hand, the antigens in PNSs have an intracellular location and are targeted by antibodies such as the anti-Yo and the anti-Hu antibodies [66,67,68]. In this review, we will also focus on epilepsy associated with AE, and the important individual disease will be introduced later in this paper.

It is also worth mentioning that the incidence of seizure disorders in patients with multiple sclerosis has been reported to exceed their incidence in the general population. Several studies have reported seizures occurring at the onset of multiple sclerosis [69]. The increased risk of seizures for patients with multiple sclerosis may reflect the effects of inflammation, which provides a theoretical basis for the application of immunomodulation to the treatment of seizure disorders.

## 5. Involvement of Ion Channels and the Immune System in Epileptogenesis and Epilepsy: Some Examples

Molecular studies of epileptogenesis and epilepsy have demonstrated that specific ion channels play an important role in both genetic and acquired forms of epilepsy [70,71,72], especially voltage-gated sodium channels [73,74,75,76,77]. The ionic mechanisms underlying burst-firing behavior in neurons are not fully understood, although sodium channels have been found to be significantly involved [72,78]. Different types of sodium channels expressed in both glutamatergic and GABAergic cell types might play unequal roles in neuronal excitability, the enhancement of synaptic potentials, the generation of subthreshold oscillations, the facilitation of repetitive firing, and the prolongation of depolarized potentials [73,79].

We previously documented that an immunomodulatory drug (glatiramer acetate) attenuated acute and chronic excitotoxicity in an animal model of epilepsy [21], with the involvement of a sodium-channel modulating effect. The interaction between sodium channel modulation, which has an earlier onset, and immunomodulation, which occurs later, is worth investigating further. Consistent with this is the fact that rotenone, an inhibitor of mitochondrial respiration and a producer of proinflammatory cytokines, has been shown to reduce paired-pulse ratios at mossy fiber-CA3 synapses, indicating a high likelihood that neurotransmitters are released, consequently exacerbating seizures [80]. Our study on hippocampal neurons showed the effect of rotenone on changes in burst firing patterns with the emergence of subthreshold potentials [81], involving sodium channels, calcium-activated chloride channels, and ATP-sensitive potassium channels. Furthermore, in our most recent study, we demonstrated that zingerone, which is widely recognized as having potent anti-inflammatory properties, produced effects on multiple ionic currents (both transient and persistent sodium currents and L-type calcium currents), as well as concerted ionic effects, may significantly impact the functional activities of neuronal cells [82]. Another recent study reported that zingerone attenuates status epilepticus by blocking hippocampal neurodegeneration via the regulation of redox imbalance, inflammation, and apoptosis [83].

Generalized epilepsy with febrile seizures plus (GEFS^+^) is caused by missense mutations in Na_V_1.1 channels. Furthermore, familial febrile seizures are caused by mild loss-of-function mutations in Na_V_1.1 channels, which are involved in febrile seizures associated with vaccination [22,84,85]. In addition, calmodulin, a small protein acting as a signal transducer that regulates neuronal plasticity, muscle contraction, and immune response [86], modulates the voltage-gated sodium-channel gating process, alters the sodium current density, and regulates the trafficking and expression of sodium channel proteins. Many mutations in the calmodulin-binding IQ domain give rise to diseases, including epilepsy [23].

As mentioned earlier, mTOR is an evolutionarily conserved serine/threonine kinase that plays a central role in integrating environmental cues in the form of growth factors, amino acids, and energy. It is now considered to be a central regulator of immune responses [87]. Mutations along the mTOR signaling pathway can indirectly affect the expression level and activity of ion channels that determine neuronal firing rate, neural activity patterns, and neuronal network activity [88]. The mTOR signaling pathway is considered one of the disease mechanisms that underlie monogenic epilepsies (i.e., mTORopathy) [89]. Both gain- and loss-of-function mutations of ion channels, synaptic proteins, and signaling molecules that are located along the mTOR pathway have been linked to this imbalance of network excitability [90]. Furthermore, in in vitro studies, mTOR was found to regulate intrinsic neuronal excitability by increasing the expression of large-conductance calcium-activated potassium channels (BK channels) [91].

The PI3K/AKT/mTOR pathway is an intracellular signaling pathway that is important in regulating the cell cycle. The PI3K/AKT pathway has a natural inhibitor called phosphatase and tensin homolog (PTEN), the function of which is to limit proliferation in cells. The NMDA receptor recruits PTEN to the postsynaptic terminal and decreases AMPA receptor-mediated responses [92]. In addition, PTEN knockdown has been shown to directly alter the properties of AMPA receptors [93]. On the other hand, mTOR inactivation has been shown to increase the expression of potassium channels (Kv1.1 and Kv1.2) and regulate the activity of NMDA receptors, which control the influx of Ca^2+^ into the cell and alter mTOR activity [94]. Thus, cellular excitability is maintained because the changes in ion channels and mTOR activity are balanced. A study by Nguyen and Anderson [95] revealed a link between ion channels and the mTOR pathway in PTEN KO mice that was associated with an increase in the expression of hippocampal Kv1.1 protein, which was normalized by the inhibition of mTOR by rapamycin. Therefore, it is possible that mTOR is capable of altering the translation and activity of a variety of ion channels in neurons, thus regulating excitability in neuronal networks [90]. Interestingly, the immunosuppressor everolimus has been shown to inhibit mTOR signaling by reducing the phosphorylation of downstream mTOR effects, which suggests that it has potential antiepileptogenic properties [14,15,16]. Whether the modulation of the mTOR pathway and immune responses, in terms of antiepileptogenesis effects, can lead to a new stage in the treatment of different forms of epilepsy remains to be determined.

## 6. Clinical Aspects of AE

Patients with AE usually present with subacute mental changes and psychiatric symptoms in conjunction with seizures, focal neurological signs, CSF pleocytosis, or changes in focal brain images featuring the finding of encephalitis [96]. In pediatric patients, the onset of the disease ranges from acute to subacute, and many patients clinically present with a focal or diffuse neurological deficit and drug-resistant epileptic seizures [97]. A systematic review of retrospective studies found an estimated efficacy of 10.7% when AEDs were used to treat 139 AE patients [98]. In another study, the prevalence of neuronal surface autoantibodies in patients with chronic epilepsy without a definite etiology was estimated at between 3% and 21% [66]. The possibility that such autoantibodies play a significant role was bolstered by a population-based study carried out in Olmsted County, USA [99]. The authors reported an increase in the incidence rate of AE from 0.4/100,000 person years in 1995–2005 to 1.2/100,000 person years in the following decade—a change that was attributed to the increased detection of autoantibodies in laboratory testing. In the same study, the prevalence of AE was shown to be higher among African Americans than Caucasians, with 38.3/100,000 and 13.7/100,000 cases, respectively. Compared with antibody activity in PNSs, autoantibodies target epitopes on the surface of the neuronal cells of patients with AE and are therefore thought to play a direct role in pathogenesis. Administering immunotherapy to AE patients has produced good treatment outcomes by removing these antibodies [65,66]. Information from a prospective registry revealed that more than 70% of adult patients with AE and new-onset seizures who had undergone immunotherapy achieved seizure remission after six months, and two-thirds of the remaining patients achieved remission after the addition of second-line therapy with rituximab [100]. In another example, a nationwide cohort study conducted in the Netherlands used both immunotherapy and AEDs to treat AE patients [101]. In total, 89% of these patients reached seizure freedom, with 53% of them becoming seizure-free immediately after undergoing immunotherapy and 14% after taking AEDs.

## 7. Antibodies against Surface Epitopes 

### 7.1. NMDA Receptor (NMDAR) Encephalitis

The NMDA receptor (NMDAR) is an ion-channel receptor protein in neurons. It has glutamate and glycine binding sites [102] and is involved in the long-term potentiation of synaptic plasticity [103]. NMDAR encephalitis usually affects young women, and the onset of clinical symptoms is accompanied by psychotic disorders, such as hallucinations, delusions, agitation, and bizarre behavior. The patients may eventually develop seizures, together with movement disorders, such as dystonia, dyskinesia, and choreoathetosis, with the possibility of autonomic dysfunctions being present as well [104]. The seizures are usually focal and nonconvulsive, but they may evolve over time into refractory status epilepticus if early treatment is not received [105] (Table 1).

The production of autoantibodies can be triggered by having previously suffered from HSV encephalitis [52], as well as ovarian teratoma [11] associated with PNSs. For example, nearly 60% of women aged 18–45 with NMDA encephalitis in one study were found to have ovarian teratoma [66]. Serological tests have shown IgG antibodies to be reactive to the GluN1 (NR1) subunit of the NMDAR receptor [106]. However, anti-NMDAR antibodies are more easily detected in CSF than in serum. As mentioned earlier, immunotherapy administered in conjunction with steroids, intravenous immunoglobulin (IVIg), and plasma exchange is the treatment of choice for anti-NMDAR encephalitis. If this fails, rituximab and cyclophosphamide are possible alternative therapies [104].

### 7.2. Leucine-Rich Glioma-Inactivated Protein 1 (LGI1) Encephalitis

The LGI1 protein is secreted by neurons and is highly expressed in the CNS, especially in the hippocampus [107]. This protein binds to presynaptic metallopeptidase protein 23 (ADAM23) and postsynaptic ADAM22 to modulate AMPA receptors, voltage-gated potassium channel (VGKC) currents, and synaptic neurotransmission/plasticity [108,109]. The LGI1 protein was once thought to be the autoantigen to VGKC [110]. Indeed, antibodies against LGI1 and those against contactin-associated protein-like 2 (Caspr2) can be seen in VGKC antibody-positive patients [109]. In addition to mutations in the LGI1 gene causing rare autosomal dominant lateral temporal lobe epilepsy (ADLTE) [111], LGI1 encephalitis, which usually occurs in men older than 60 years of age [112,113,114], is characterized by repetitive, brief faciobrachial dystonic seizures (FBDSs) [112], as well as behavioral changes and disorientation. This type of dystonic, focal motor seizure primarily affects the arm and the ipsilateral side of the face. Its clinical presentation is often misdiagnosed as a psychogenic disorder or various types of movement disorders. Without early recognition and the prompt initiation of immunotherapy, patients suffer cognitive dysfunctions and behavioral changes as sequelae [113,114]. It is possible, by and large, to get control over the seizures within two months with steroid treatment (Table 1).

### 7.3. Anti-GABA-B Receptor (Anti-GABA_B_R) Encephalitis

Anti-GABA_B_R encephalitis is a common type of AE, in addition to NMDAR encephalitis and LGI1 encephalitis. More than half of Anti-GABA_B_R patients suffer from a paraneoplastic syndrome caused by small cell lung cancer. The types of seizures involved may be complex partial or secondarily generalized seizures, or the seizures may evolve into status epilepticus, as is encountered in most cases of limbic encephalitis. Furthermore, anti-GABA_B_R encephalitis is sometimes associated with memory loss and insomnia [115,116,117]. It is prevalent predominantly in males older than 40 years of age (median age: 61) [64]. Although anti-GABA_B_R antibodies are present in limbic encephalitis associated with small cell lung cancer, they have rarely been seen in serological evaluations of patients with small cell lung cancer [117,118]. These findings suggest that the antibody itself has a pathogenic effect in the absence of any underlying tumors (Table 1).

### 7.4. Anti-GABA-A Receptor (Anti-GABA_A_R) Encephalitis

The GABA_A_ receptor is a ligand-gated chloride channel that mediates inhibitory neurotransmission. Impairment of GABA_A_ receptor-mediated inhibition is one of the basic mechanisms of neuronal hyperexcitability. Mutations in GABRG_2_, which encodes the ϒ2 subunit of GABA_A_ receptors, and GABRA_1_, which encodes the α1 subunit, have been associated with idiopathic generalized epilepsy [119]. Furthermore, GABA_A_ receptor autoantibodies cause the downregulation of GABA_A_ receptors, leading to AE. This form of encephalitis is usually severe and is characterized by altered behavior, cognitive impairment, disturbance of consciousness, and movement disorders. Seizures are a frequent symptom, and patients may present with fulminant seizure activity or refractory status epilepticus. Brain MRIs of such patients have shown hyperintensity on a T2/fluid-attenuated inversion recovery (FLAIR) MRI sequence in multiple cortical and subcortical areas [120,121,122]. Immunotherapy is the mainstay of treatment in such cases, as it is for other types of limbic encephalitis [120,121] (Table 1).

### 7.5. Anti-Caspr2 Encephalitis

Contactin-associated protein-like 2 (Caspr2) is a cell adhesion molecule in the juxtaparanodal region of myelinated axons in the CNS and the peripheral nervous system (PNS) [113,123]. The juxtaparanodal complex is involved in organizing the distribution of potassium channels. Patients with anti-Caspr2 antibodies present with various syndromes involving increased excitability in the CNS and PNS. Anti-Caspr2 encephalitis has been associated with cognitive decline, depression, delusions, and various types of recurrent seizures, including focal, generalized, and nonconvulsive status epilepticus [124,125,126]. Increased excitability in the PNS has been observed to lead to pain and neuromyotonia [127]. This type of encephalitis occurs in elderly men, with a median age at onset of 66. Recourse to immunotherapy, either with a combination of IVIg, steroids, and plasma exchange or as a second-line treatment consisting of cyclophosphamide or rituximab, was shown to achieve full recovery in 40% of patients and partial recovery in 52% of patients [126]. After a median follow-up period of 3 years, 73% of the patients showed favorable clinical outcomes (scores ≤2 on the modified Rankin scale). However, up to 25% of the patients experienced a relapse of symptoms after one year. Notably, Caspr2 antibody-associated syndromes were diagnosed in some patients during the relapse stage, indicating the diagnostic difficulties posed by this disease (Table 1).

### 7.6. Dipeptidyl-Peptidase-Like Protein 6 (DPPX) Antibodies

The dipeptidyl-peptidase-like protein 6 (DPPX) epitope is a cell surface auxiliary subunit of the Kv4.2 potassium channel [128]. The neuropsychiatric manifestations of DPPX encephalitis include agitation, hallucinations, myoclonus, tremors, and seizures [128,129]. Progressive encephalomyelitis with rigidity and myoclonus (PERM) has also been observed [130]. Noninfectious diarrhea and weight loss sometimes precede the occurrence of neurological symptoms [131], which raises the possibility that antibodies act against the epitope in both the CNS and the gut (Table 1).

### 7.7. α-Amino-3-hydroxy-5-methyl-4-isoxazolepropionic Acid (AMPA) Receptor Antibody

The AMPA receptors, together with the NMDA and kinetic receptors, are the main mediators of excitatory neurotransmission. A difference between them is that the AMPA receptors are involved in fast synaptic signal transduction, whereas the NMDA receptors are associated with slow transduction and long-term synaptic potentiation [132]. The AMPA receptor antibodies target the GluR1 and GluR2 subunits and cause direct antibody-mediated pathologies [133,134], resulting in symptoms typical of limbic encephalitis, such as anterograde and retrograde memory impairments, mood changes, psychotic symptoms, and seizures [134]. However, a wide range of clinical symptoms have been noted. Some patients present with sleep disorders, signs of cerebellar dysfunction, nystagmus, speech dysfunctions, and even fulminant encephalitis symptoms culminating in a coma [135]. This type of encephalitis has been found to manifest as paraneoplastic limbic encephalitis, which may be associated primarily with small cell lung cancer and thymoma [134]. The response to immunotherapy is modest, and the presence of other onconeural antibodies and paraneoplastic syndromes is a predictor of poor outcomes [134] (Table 1).

## 8. Antibodies of Intracellular Epitopes 

### 8.1. Glutamic Acid Decarboxylase (GAD65) Antibody

The glutamic acid decarboxylase (GAD65) antibodies are a biomarker of type I diabetes. A low GAD65 antibody titer does not significantly correlate with autoimmune neurological diseases, which has been confirmed by observations of patients with isolated type I diabetes and of healthy persons [136,137], whereas high GAD65 antibody titers (>20 nmol/L in CSF or in serum) are associated with autoimmune neurological diseases. Women are more frequently affected, with such diverse clinical symptoms as autoimmune epilepsy, stiff-person syndrome, cerebellar ataxia, limbic encephalitis, brainstem dysfunction, and PERM [138,139]. A particularly high GAD65 antibody titer (>1000 U l/mL) was noted in 5.4% of the members of a group of 112 patients with adult-onset focal epilepsy with unknown etiologies that was resistant to treatment [140]. Furthermore, in a recent survey of 212 patients, a GAD65 antibody titer of more than 500 nmol/L was associated with poor outcomes [139]. Among the clinical symptoms that were manifested in these patients, epilepsy was least responsive to immunotherapy, whereas stiff-person syndrome responded more favorably to treatment (Table 1).

### 8.2. Antineuronal Nuclear Antibody Type 1 (ANNA-1, Anti-Hu)

The antineuronal nuclear antibody type 1 (ANNA-1) antibodies are associated with small cell lung cancer. HuD-specific T cells triggered by cytokines produced by tumor cells are thought to play a pathogenic role in paraneoplastic syndromes [61]. The symptoms of ANNA-1 autoimmunity encompass both peripheral sensory neuropathy and autonomic neuropathy, and they have been shown to be especially involved in gastrointestinal motility disorders [141,142]. Patients with this ANNA-1 encephalitis can present with seizures or epilepsia partialis continua [143,144], and EEG results have shown temporal or extratemporal slow waves or epileptiform discharges [143]. In such cases, the outcome of immunosuppressive treatments is usually not satisfactory [145] (Table 1).

### 8.3. Antineuronal Antibodies Ma1 and Ma2

Patients with isolated Ma2 antibodies (also known as Ta) usually present with isolated or combined limbic, brainstem, or diencephalic encephalitis. Seizure types include bilateral tonic–clonic and focal with unawareness [8]. Patients who are seropositive for Ma-2 antibodies tend to be young men with underlying testicular germ cell tumors. This group of patients shows favorable treatment outcomes [8,146]. A link between anti-Ma antibodies and limbic encephalitis has also been found in breast cancer patients [147]. Finally, coexisting Ma1 antibodies tend to occur in older men with small cell lung cancer or bladder cancer, and they usually respond unfavorably to treatment [148] (Table 1).

### 8.4. Collapsin Response Mediator Protein-5

The collapsin response mediator protein-5 (CRMP-5) antibodies are a biomarker of paraneoplastic syndromes associated with small cell lung cancer or thymoma [149]. Although their autoimmunity may cause paraneoplastic encephalitis, it is not always confined to the limbic system. The clinical presentations can be found to affect both the CNS and the PNS, with various symptoms manifested, including chorea, cerebellar ataxia, cranial nerve neuropathy, myelopathy, and sensorimotor polyneuropathy/polyradiculopathies [105,150,151]. The seizures that have been reported are focal seizures with awareness or unawareness. The treatment of choice is a combination of removal of the underlying tumor and early administration of immunosuppressive therapy [152] (Table 1).

**Table 1:** Antibodies against intracellular or surface epitopes in autoimmune epilepsy.

Intracellular Epitopes				
Target of Antibodies	Clinical Symptoms/Syndromes	Functions of Targets	Underlying Tumors	Ref.
GAD65	AE, LE, stiff-person syndrome, ataxia, type I diabetes	Synthesis of GABA	Lung, thymoma	[136,137,138,139]
ANNA-1 (anti-Hu)	AE, epilepsia partialis continua, sensory and autonomic neuropathy	HuD-specific T cells triggered by cytokines	SCLC	[61,141,142,143,144]
Ma1 and Ma2 (Ta)	LE, brainstem Encephalitis, tonic-clonic or focal unawareness seizure	RNA transcription regulation	SCLC, bladder, testicular germ cell, breast cancer	[8,147]
CRMP-5	AE, chorea, ataxia, cranial neuropathy, sensorimotor polyneuropathy	Guides the developing axons in the nervous system	SCLC, thymoma	[105,150,151]
**Surface Epitopes**				
NMDA receptor	LE, movement disorders, psychosis	Long-term potentiation of synaptic plasticity	Ovarian teratoma	[66,103,104,105]
LGI1 protein	LE, faciobrachial dystonic seizure, neuromyotonia	Binds to presynaptic ADAM23 and postsynaptic ADAM22 to modulate AMPA receptors, VGKC currents, and synaptic neurotransmission/plasticity	Thymoma (rare)	[108,109,110,111,112]
GABA_B_ receptor	LE, seizures, memory loss	Mediates slow and prolonged inhibitory action	SCLC	[115,116,117,118]
GABA_A_ receptor	Seizures, psychosis, cognitive impairment	Ligand-gated chloride channel that mediates fast inhibitory transmission	SCLC, thymoma, NHL	[119,120,121,122]
Caspr2	Seizures, cognitive decline, neuromyotonia, neuropathic pain	Cell adhesion molecule in the juxtaparanodal complex organizes the distribution of potassium channels	Thymoma (rare)	[113,124,125,126,127]
DPPX	Seizures, hallucination, PERM, prodromal diarrhea	Cell-surface auxiliary subunit of the Kv4.2 potassium channel	Lymphoma (rare)	[128,129,130,131]
AMPA receptor	LE, seizures, memory loss	Mediators of excitatory neurotransmission	SCLC, breast/ovarian cancer	[133,134]

AMPA: α-Amino-3-hydroxy-5-methyl-4-isoxazolepropionic acid; AE: autoimmune encephalitis; ANNA-1: antineuronal nuclear antibody 1; Caspr2: contactin-associated protein-like 2; CRMP-5: collapsin response mediator protein-5; DPPX: dipeptidyl-peptidase-like protein 6; GABA: gamma-aminobutyric acid; GAD: glutamic acid decarboxylase; LE: limbic encephalitis; LGI1: leucine-rich glioma-inactivated protein 1; NMDA: N-methyl-D-aspartate; NHL: non-Hodgkin lymphoma; SCLC: small cell lung cancer; PERM: progressive encephalomyelitis with rigidity and myoclonus; SCLC: small cell lung cancer.

## 9. Miscellaneous Antibodies

### 9.1. New-Onset Refractory Status Epilepticus

New-onset refractory status epilepticus (NORSE) usually affects children or young adults, for two-thirds of whom flu-like symptoms precede the onset of refractory status epilepticus [153]. It is possible that the condition of previously healthy persons presenting with a first-ever seizure may evolve into refractory status epilepticus. In a retrospective analysis of 130 patients with NORSE, the most common etiologies (37%) were autoimmune and paraneoplastic encephalitis [154], indicating that a proportion of these cases involved immune-mediated etiologies. Immunotherapy has shown only modest efficacy, whereas a ketogenic diet has been suggested as an alternative therapy [155].

### 9.2. Predictors of Autoimmune Epilepsy and Responses to Immunotherapy

As we come to understand the role of immunity in the pathogenesis of epilepsy, the question arises as to how and when to undertake serological testing for antibodies in patients with epilepsy of unknown etiologies. Dubey et al. proposed a predictive model of antibody prevalence in epilepsy (APE) [156] and updated APE2 score [157] based on clinical characteristics (see Table 2). In their retrospective analysis of 262 patients with epilepsy of unknown etiologies, CNS-specific antibodies were detected in 44 patients (16.8%). An APE or APE2 score equal to or higher than 4 has been associated with of the presence of neuron-specific antibodies [156]. For patients with epilepsy of unknown etiology who have an APE2 score equal to or higher than 4, despite a thorough workup and negative autoantibody evaluation, a diagnostic trial of immunotherapy should be considered. An APE2 score ≥ 7 had specificity of 100% for an autoimmune etiology of epilepsy. Response to immunotherapy in epilepsy (RITE) or updated RITE2 score (see Table 3) are also believed to predict therapeutic outcomes. An RITE2 score equal to or higher than 7 indicates a favorable seizure outcome as a result of immunotherapy [156,157].

First-line immunotherapy includes high-dose corticosteroids (empiric treatment with intravenous methylprednisolone at a dose of 1 g per day for 3–7 days), IVIg at a dose of 2 g/kg over 2–5 days, and plasma exchange (5–10 sessions every other day) [158,159]. Clinicians may consider using combined first-line therapies if the clinical picture is severe. If there is no meaningful response to the first-line therapy after 2–4 weeks, the addition of a second-line agent can improve the outcome. Second-line agents include rituximab (common rituximab dosing regimens include 375 mg/m^2^ weekly for 4 weeks or two doses of 1000 mg 2 weeks apart) or cyclophosphamide (common dosing regimens of cyclophosphamide include 600–1000 mg/m^2^ in a 3–6 monthly cycles). Of note, body surface area (BSA, m^2^) equals the square root of (height (cm) × weight (kg)/3600) [158].

In terms of side effects of immunotherapy, clinicians should be careful in using corticosteroids in patients with hypertension or diabetes. Common side effects of IVIg include chillness, fever, flu-like symptoms, headache, soreness, and skin reactions. More severe reactions include inflammation of organs, such as hepatitis, myocarditis, meningoencephalitis, and pancreatitis, as well as thrombotic effect [160,161]. Delayed thrombotic effect was found to affect 1% to 16.9% of patients receiving IVIg [162,163]. In addition, increased bleeding risk and volume shifts [158] related to plasma exchange have been observed. In the second-line immunotherapy, clinicians should be aware that the leukopenia are common reactions in patients receiving rituximab and cyclophosphamide therapy [164,165,166].

Another question arising in this context that deserves further investigation is whether immunotherapy is warranted for treatment of epilepsy patients with detected neuronal antibodies, for treatment of patients with pharmacoresistant epilepsy (PRE), or for both. In a retrospective immunotherapeutic trial with PRE patients who had neuronal surface antibodies, 62% of the patients responded to immunotherapy, and 34% achieved seizure freedom [24]. On the other hand, another study found that patients suspected of having autoimmune limbic encephalitis without neuronal antibodies benefitted from immunotherapy [167]. Despite being limited, this evidence indicates that the decision to administer immunotherapy to patients who have tested positive for neuronal antibodies depends on judgements in response to clinical signs and symptoms of concomitant AE, rather than solely on the presence of antibodies in patients experiencing new-onset seizures [167,168]. Another prospective study predicted neuronal surface autoantibodies in new-onset focal epilepsy and evaluated the results of immunotherapy. In this cohort, 10.5% of the patients were antibody-positive, and 40% were diagnosed with AE. The authors identified six features that were highly predictive of the presence of antineuronal antibodies: age over 54 years, ictal piloerection, self-reported lowered moods, MRI-recorded changes in the limbic system, reduced attention, and the absence of conventional epilepsy risk factors [159]. Nearly 80% of the patients with detectable autoantibodies who showed no evidence of AE had good long-term outcomes (modified Rankin score = 0) despite not being given immunotherapy. Notably, the outcomes for this group of patients were better than those for patients with confirmed autoantibody-mediated encephalitis who had received immunotherapy. These findings echo the aforementioned importance of treating the patients rather than simply treating the laboratory findings.

## 10. Conclusions

Epilepsy is a multifaceted condition involving complex etiologies, including immunological alterations. The ILAE’s classification of seizures and epilepsies has added an immune origin as one of the six etiologies of epilepsy, and numerous neuronal autoantibodies have been found in patients with epilepsy. The role of immunity and its interaction with ion channels in epilepsy is gradually receiving increasing attention. Patients with autoimmune encephalitis often present with severe seizures or status epilepticus. Early recognition of immune-mediated epilepsy is important, especially in cases of pharmacoresistant epilepsy and in the presence of signs of encephalitis, as early intervention with immunotherapy is potentially promising. Clinical trials examining the efficacy of immune modulation for treatment of different types of epilepsy warrant attention in future research.

## Figures and Tables

**Table 2:** APE and APE2 score and components [156,157].

APE Score and Components	
Clinical Characteristics	Score
New-onset, rapidly progressive mental status changes that developed over 1–6 weeks or new-onset seizure activity (within 1 year of evaluation)	1
Neuropsychiatry changes; agitation, aggressiveness, emotional lability	1
Autonomic dysfunction (sustained atrial tachycardia or bradycardia, orthostatic hypotension (≥20 mm Hg fall in systolic pressure or ≥10 mm Hg fall in diastolic pressure within 3 min of standing), hyperhidrosis, persistently labile blood pressure, ventricular tachycardia, or cardiac asystole)	1
Viral prodrome (rhinorrhea, sore throat, low-grade fever) in the absence of underlying malignancy	2
Facial dyskinesias or faciobrachial dystonic movements	2
Refractory seizure	2
CSF: signs of inflammation (elevated CSF protein >50 mg/dL and/or lymphocytic pleocytosis >5 cells/dL if the total number of CSF RBC is <1000 cells/dL ^a^	2
Brain MRI findings indicate limbic encephalitis (medial temporal T2/FLAIR signal changes) ^a^	2
Underlying malignancy (excluding cutaneous squamous cell carcinoma and basal cell carcinoma)	2
a: No brain MRI or CSF analyses were scored zero.	Total max: 15
**APE2 Score and Components**	
**Clinical Characteristics**	**Score**
New-onset, rapidly progressive mental status changes that developed over 1–6 weeks or new-onset seizure activity (within 1 year of evaluation)	1
Neuropsychiatry changes: agitation, aggressiveness, emotional lability	1
Autonomic dysfunction (sustained atrial tachycardia or bradycardia, orthostatic hypotension, hyperhidrosis, persistently labile blood pressure, ventricular tachycardia, cardiac asystole, or gastrointestinal dysmotility) *	1
Viral prodrome (rhinorrhea, sore throat, low-grade fever) in the absence of underlying systemic malignancy within 5 years of neurological symptom onset	2
Faciobrachial dystonic seizures	3
Facial dyskinesias in the absence of faciobrachial dystonic seizures	2
Seizure refractory to at least two antiseizure medications	2
CSF signs of inflammation # (elevated CSF protein >50 mg/dL and/or lymphocytic pleocytosis >5 cells/dL if the total number of CSF RBC is <1000 cells/dL	2
Brain MRI suggesting encephalitis (T2/FLAIR hyperintensity restricted to one or both medial temporal lobes or multifocal in gray matter, white matter, or both compatible with demyelination or inflammation)	2
Systemic cancer diagnosed within 5 years of neurological symptom onset (excluding cutaneous squamous cell carcinoma, basal cell carcinoma, brain tumor, cancer with brain metastasis)	2
* Scored only if no history of autonomic dysfunction prior to onset of suspected autoimmune syndrome and the autonomic dysfunction not attributable to medications, hypovolemia, plasmapheresis, or infection # Patients scored zero if brain MRI or CSF analysis not performed	Total max: 18

Ab, antibody, AMPA-R = amino-3-hydroxy-5-methyl-4-isoxazolepropionic acid; CASPR-2 = contactin-associated protein 2; DPPX = dipeptidyl-peptidase-like protein 6; FLAIR = fluid-attenuated inversion recovery; GABA_A_R = γ-aminobutyric acid A receptor; GABA_B_R = γ-aminobutyric acid B receptor; GAD-65, glutamic acid decarboxylase 65; GFAPα = glial fibrillary acidic protein; LGI1 = leucine-rich glioma-inactivated protein 1; NMDA-R = N-methyl-d-aspartate receptor. AMPA-R = amino-3-hydroxy-5-methyl-4-isoxazolepropionic acid; CASPR-2 = contactin-associated protein 2; DPPX = dipeptidyl-peptidase-like protein 6; FLAIR = fluid-attenuated inversion recovery; GABA_A_R = γ-aminobutyric acid A receptor; GABA_B_R = γ-aminobutyric acid B receptor; GFAPα = glial fibrillary acidic protein; LGI1 = leucine-rich glioma-inactivated protein 1; MOG = myelin oligodendrocyte glycoprotein; mGluR1 = metabotropic glutamate receptor 1; mGluR5 = metabotropic glutamate receptor 5; NMDA-R = N-methyl-d-aspartate receptor.

**Table 3:** RITE [156] and RITE2 score [157]: includes all the components of APE and APE2 score and two additional variables: initiation of immunotherapy within 6 months of symptom onset and presence of membrane autoantibody.

Clinical Characteristics	Score
Rapid progressive mental change within 6 weeks or new-onset seizure within one year	1
Neuropsychiatry symptoms: agitation, aggressiveness, emotional lability	1
Autonomic dysfunction (sustained atrial tachycardia or bradycardia, orthostatic hypotension (≥20 mm Hg fall in systolic pressure or ≥10 mm Hg fall in diastolic pressure within 3 min of standing), hyperhidrosis, persistently labile blood pressure, ventricular tachycardia, or cardiac asystole)	1
Viral prodrome (rhinorrhea, sore throat, low-grade fever) in the absence of underlying malignancy	2
Facial dyskinesias or faciobrachial dystonic movements	2
Refractory seizure	2
CSF: signs of inflammation (elevated CSF protein >50 mg/dL and/or lymphocytic pleocytosis >5 cells/dL if the total number of CSF RBC is <1000 cells/dL ^a^	2
Brain MRI findings indicate limbic encephalitis (medial temporal T2/FLAIR signal changes) ^a^	2
Underlying malignancy (excluding cutaneous squamous cell carcinoma, basal cell carcinoma)	2
Initiation of immunotherapy within 6 month of symptom onset	2
Presence of neural plasma membrane autoantibody (e.g., NMDA-R Ab, GABA_A_-R Ab, GABA_B_-R AMPA-R Ab, DPPX, GAD-65, LGI-1 Ab, or CASPR-2 Ab)	2
a: No brain MRI or CSF analyses were scored zero.	Max: 19
New-onset, rapidly progressive mental status changes that developed over 1–6 weeks or new-onset seizure activity (within 1 year of evaluation)	1
Neuropsychiatry symptoms: agitation, aggressiveness, emotional lability	1
Autonomic dysfunction (sustained atrial tachycardia or bradycardia, orthostatic hypotension, hyperhidrosis, persistently labile blood pressure, ventricular tachycardia, cardiac asystole, or gastrointestinal dysmotility) *	1
Viral prodrome (rhinorrhea, sore throat, low-grade fever) only to be scored in the absence of underlying malignancy within 5 years of neurological symptom onset	2
Faciobrachial dystonic seizures	3
Facial dyskinesias in the absence of faciobrachial dystonic seizures	2
Seizure refractory to at least two antiseizure medications	2
CSF signs of inflammation # (elevated CSF protein >50 mg/dL and/or lymphocytic pleocytosis >5 cells/dL if the total number of CSF RBC is <1000 cells/dL	2
Brain MRI suggesting encephalitis (T2/FLAIR hyperintensity restricted to one or both medial temporal lobes or multifocal in gray matter, white matter, or both compatible with demyelination or inflammation)	2
Systemic cancer diagnosed within 5 years of neurological symptom onset (excluding cutaneous squamous cell carcinoma, basal cell carcinoma, brain tumor, cancer with brain metastasis)	2
Initiation of immunotherapy within 6 month of symptom onset	2
Neural plasma membrane autoantibody detected (NMDA-R, GABA_A_R, GABA_B_R, AMPA-R, DPPX, mGluR1, mGluR5, LGI1, CASPR-2, neurexin-3α, MOG)	2
* Scored only if no history of autonomic dysfunction prior to onset of suspected autoimmune syndrome and the autonomic dysfunction is not attributable to medications, hypovolemia, plasmapheresis, or infection # Patients scored zero if brain MRI or CSF analyses not performed	Max: 22

Ab, antibody, AMPA-R = amino-3-hydroxy-5-methyl-4-isoxazolepropionic acid; CASPR-2 = contactin-associated protein 2; DPPX = dipeptidyl-peptidase-like protein 6; FLAIR = fluid-attenuated inversion recovery; GABA_A_R = γ-aminobutyric acid A receptor; GABA_B_R = γ-aminobutyric acid B receptor; GAD-65, glutamic acid decarboxylase 65; GFAPα = glial fibrillary acidic protein; LGI1 = leucine-rich glioma-inactivated protein 1; NMDA-R = N-methyl-d-aspartate receptor; PCA-1 = Purkinje cell cytoplasmic antibody type 1; PCA-2 = Purkinje cell cytoplasmic antibody type 2; AMPA-R = amino-3-hydroxy-5-methyl-4-isoxazolepropionic acid; CASPR-2 = contactin-associated protein 2; DPPX = dipeptidyl-peptidase-like protein 6; FLAIR = fluid-attenuated inversion recovery; GABA_A_R = γ-aminobutyric acid A receptor; GABA_B_R = γ-aminobutyric acid B receptor; GFAPα = glial fibrillary acidic protein; LGI1 = leucine-rich glioma-inactivated protein 1; MOG = myelin oligodendrocyte glycoprotein; mGluR1 = metabotropic glutamate receptor 1; mGluR5 = metabotropic glutamate receptor 5; NMDA-R = N-methyl-d-aspartate receptor.

## Data Availability

Not applicable.

## Abbreviations

ADAM23	a disintegrin and metalloprotease domain 23
ADLTE	autosomal dominant lateral temporal lobe epilepsy
AE	autoimmune encephalitis
AEDs	antiepileptic drugs
AMPA	α-amino-3-hydroxy-5-methyl-4-isoxazolepropionic acid
ANNA-1	antineuronal nuclear antibody type 1
APE	antibody prevalence in epilepsy
BBB	blood-brain barrier
Caspr2	contactin-associated protein-like 2
CNS	central nervous system
CRMP-5	collapsin response mediator protein-5
DPPX	dipeptidyl-peptidase-like protein 6
FBDSs	faciobrachial dystonic seizures
FLAIR	fluid-attenuated inversion recovery
GABA	ϒ-aminobutyric acid
GAD65	glutamic acid decarboxylase
GEFS+	generalized epilepsy with febrile seizures plus
HMGB1	high-mobility group box protein 1
HS	hippocampal sclerosis
HSV	herpes simplex virus
IL-1	interleukin-1
ILAE	International League Against Epilepsy
IFNγ	interferon gamma
IVIg	intravenous immunoglobulin
LE	limbic encephalitis
LGI1	leucine-rich glioma-inactivated protein 1
LPS	lipopolysaccharide
MOG	myelin oligodendrocyte glycoprotein
mTOR	mammalian target of rapamycin
NMDA	N-methyl-D-aspartate
NMDAR	NMDA receptor
NORSE	New-onset refractory status epilepticus
NR1	GluN1
PERM	progressive encephalomyelitis with rigidity and myoclonus
PET	positron emission tomography
PNS	peripheral nervous system
PNSs	paraneoplastic neurological syndromes
PRE	pharmacoresistant epilepsy
PTEN	phosphatase and tensin homolog
RITE	response to immunotherapy in epilepsy
TLE	temporal lobe epilepsy
TLR4	toll-like receptor 4
TNFα	tumor necrosis factor alpha
VGKC	voltage-gated potassium channel
